# The genetics of cancer heterogeneity and mesothelioma

**DOI:** 10.3389/fonc.2026.1744382

**Published:** 2026-03-16

**Authors:** Lucian R. Chirieac, Ritu Gill, Richard Attanoos

**Affiliations:** 1 Department of Pathology, Mount Sinai Hospital, New York, NY, United States; 2 Columbia University Irving Medical Center, Vagelos College of Physicians and Surgeons, New York, NY, United States; 3 Department of Cellular Pathology, University Hospital of Wales and School of Medicine, Cardiff University, Wales, United Kingdom

**Keywords:** BAP1, germline, immunohistochemistry, mesothelioma, pathology, radiology, ALK rearrangement, cancer heterogeneity

## Abstract

Diffuse mesothelioma is an invasive cancer that originates from the cells in the smooth tissue lining (serosal membrane) that surrounds various body cavities. While most cases originate in the pleural lining of the thoracic cavity, a subset primarily involves the peritoneum or, rarely, the pericardium or the tunica vaginalis. Advances in molecular biology have established that cancer heterogeneity is common across a wide variety of histogenetically diverse neoplasms and that ‘mesothelioma’ as a disease is the same. It is increasingly evident that age, sex, and anatomic site-specific variations do exist which are often driven by recognized and recurrent mutations although a high degree of inter- and intra-tumor heterogeneity is present, and this is reviewed. Diverse patterns of disease exist with respect to clinical, radiologic, pathologic findings and these are driven by unique molecular events, the mechanisms and origin of which are increasingly determined to be due to stochastic events. Consequently, mesothelioma has not only considerable radiologic, macroscopic, and microscopic heterogeneity, but includes multiple distinct genetic entities. Most mesotheliomas are characterized by recurrent mutations in tumor suppressor genes and epigenetic regulators, including *BAP1, NF2, TP53, SETD2*, and other genes. Alterations are identified in multiple pathways in the regulation of cell-cycle, RNA processing, histone regulation, and cell growth. *BAP1* is one of the most frequently altered genes and is activated by diverse mechanisms including *BAP1* point mutations, copy number loss, inactivating structural rearrangements, and minute chromosomal deletions. Consistent with its histomorphologic heterogeneity, mesothelioma displays an impressive molecular diversity. Subsets of mesothelioma have unusual genetic alterations: genomic near-haploidization in rare pleural mesotheliomas with mutations in *TP53* and/or *SETDB1*; oncogenic *EWSR1-ATF1* fusion; *ALK* rearrangements in rare patients with peritoneal mesothelioma. In addition, germline mutations are present in a subset of patients with mesothelioma and primarily involve genes in the DNA repair and cell cycle regulation and are more common in patients who are young, with family history of mesothelioma, or with peritoneal mesothelioma. In this review, we discuss the considerable heterogeneity of mesothelioma, the diversity of radiologic and gross presentation, various morphologic features with distinctive histologies and ultimately, we individually describe subsets of tumors characterized by uncommon alterations such as germline mutations, genomic near-haploidization, *ALK* rearrangement, *ATF1* rearrangement, or *EWSR1::YY1* fusion, as well as the implications of these findings on the diagnostic workup.

## Introduction

Diffuse mesothelioma is an invasive cancer that originates from the cells in the serosal membranes that surround various body cavities (1, 2). The percentage of mesotheliomas in the United States by anatomic site is approximately 85-90% in the pleura, approximately 15% in the peritoneum (the serosal membrane covering abdominal organs), and ultimately, less than 1% combined are located in the pericardium (the membrane enclosing the heart) or the tunica vaginalis (the serosal membrane that covers the testes) (3). Additionally, the term “mesothelioma” has been used historically for both benign serosal proliferations (namely benign mesothelioma or adenomatoid tumor, and multicystic mesothelioma or peritoneal inclusion cyst)as well as for neoplasms of uncertain malignant potential, such as well-differentiated papillary mesothelioma (WDPM) reclassified as well-differentiated papillary mesothelial tumor (WDPMT) (4) in 2021 and mesothelioma-*in situ* (MIS).

Mesothelioma includes several clinicopathologic subgroups, with distinct site predilections and pathogenetic mechanisms. It is recognized that mesothelioma may demonstrate considerable clinical, radiologic, gross, and microscopic pathologic heterogeneity, as well as diverse molecular genetic abnormalities. An overview is provided of the recent discoveries on the genetics and pathogenesis of mesothelioma, as well as the implications of these findings on the diagnostic workup. A systematic literature review was conducted of recently described clinical, imaging, pathologic, and genetic features. The source data was identified via a search engine on US National Library of Medicine National Institutes of Health NCI PubMed, incorporating from initial key words ‘malignant mesothelioma’, peritoneal’, pleural’, germline,’ BAP1,’ ALK,’ and ‘radiation.’ Data was obtained from published literature of mesotheliomas in epidemiological cohorts of asbestos exposed persons, cancer registries, case-control studies, case series and case reports.

It is clear that molecular approaches, and immunohistochemical tools in mesothelioma have been established that have evolved our understanding of the molecular diversity of mesothelioma (2). This in turn has led to considerable advances in our clinical practice, and management of mesothelioma. including the application of genomic technologies and immunohistochemical markers such as BAP1, MTAP, and merlin (NF2) that are surrogates of their mutation status.

## Mesothelioma incidence and nomenclature

In the United States, pleural mesothelioma has historically affected approximately 2,500 to 3,300 patients each year since 1999, with an annual incidence of approximately 1 in 100,000 people (5–7). The 85-90% pleura mesotheliomas shows a predilection for men (M:F; 3-4:1) with a median age of 73 years (8–10). These cases typically relate to prior workplace exposures to commercial amphibole asbestos. The age distribution of mesothelioma is wide, with a minority of cases in children and young patients. These have a different etiology linked extensively to specific genetic aberrations (11–13).

## Causes of mesothelioma

In the past, the most common cause of *pleural* mesothelioma was considered to be exposure to a sufficient dose of amphibole asbestos, historically reported in 80-90% of male patients and 20-40% of female patients (5, 14–17). Asbestos exposure is less common in *peritoneal* mesothelioma, where less than 25% of *peritoneal* mesothelioma in men and 15% of *peritoneal* mesotheliomas in women are related to amphibole asbestos exposure (18–23). The percentage of non-asbestos-related mesothelioma has increased over time and is reflective of the less widespread uses of commercial amphibole asbestos (24).

Other causes of mesothelioma, pleural or peritoneal, include exposure to non-asbestos mineral fibers (25), therapeutic radiation exposure for prior malignancy (26, 27), and chronic inflammatory conditions, such as endometriosis, chronic empyema, old scars, and Crohn disease (21, 23, 28–34).

A proportion of mesothelioma do arise in the absence of clear extrinsic etiologic factors, due to stochastic replication errors and epigenetic alterations – this group are termed ‘naturally-occurring’ cancers or spontaneous/sporadic mesothelioma. Intrinsic genetic events may represent gain of function oncogenic fusions with a variety of partner genes such as structural gene rearrangements of Ewing sarcoma breakpoint region 1 (*EWSR1*) gene or fused in sarcoma (*FUS*) gene (35), and anaplastic lymphoma kinase (*ALK*) gene rearrangements, recently described in a study regarding a cohort of young women with peritoneal mesothelioma (36).

Furthermore, inherited familial mutations such as pathogenic variant germline alterations in *BAP1* and in other tumor suppressor genes have been implicated in the development of mesothelioma in a subset of approximately 12%-16% of patients (37–42). These prevalence values are not unique to patients with mesothelioma, and analyses of patients undergoing tumor-normal sequencing with cancer-specific gene panels, reported that approximately 16% of patients carried germline pathogenic or likely pathogenic variants in a gene linked to an inherited human disease (43). Depending on the patient cohort and the method used for identification of germline variants previous studies have reported a prevalence of putative pathogenic germline variants in 4.3–17.5% of cancer patients (43–46). A recent study (47) investigated a cohort of 636 patients with advanced cancer for pathogenic germline variants to explore associations between germline variants, cancer types and molecular pathways (47). Out of these patients, 17.8% (n = 113) had a pathogenic or likely pathogenic germline variant in at least one of the 168 cancer-associated genes tested.

## Clinical, macroscopic and radiologic heterogeneity of mesothelioma

Pleural mesothelioma exhibits considerable macroscopic and radiologic heterogeneity, reflective of its histopathologic diversity and clinical course.

The clinical diversity of symptomatology and physical signs in mesothelioma, in part, is reflective on its gross disease distribution, or rarely results from secondary metastatic involvement or systemic paraneoplastic disease. The vast majority of mesotheliomas grow along the serosal membranes with circumferential pleural thickening or multifocal-to-diffuse pleural nodules that display a tan-white, or gray cut surface. Invasion into adjacent structures such as diaphragm, chest wall, and pericardium, and its interlobular septa may be present. In rare cases, pleural mesothelioma presents with diffuse lung parenchymal involvement, with radiographic and gross appearances mimicking an interstitial lung disease (48–50). A limited number of pleural mesotheliomas present as spontaneous pneumothorax (51–55).

Patients vary widely in symptom onset and initial presentation - dyspnea, chest pain, weight loss, and large pleural effusions in pleural disease (56, 57); abdominal distension, pain, and ascites in peritoneal disease (58). Furthermore, the evaluation of staging and tumor burden in patients with mesothelioma in order to tailor the therapeutic choices, is challenging since tumor growth is sheet-like rather than nodular, and nodal assessment is inconsistent; this contributes to heterogeneous prognostication and treatment selection (59, 60). Although usually considered an aggressive locoregional malignancy, pleural mesothelioma shows variable metastatic behavior - including extrathoracic spread and brain metastases in a subset of cases - affecting surveillance and palliation therapeutic strategies (61, 62). Data from the SEER-Medicare database for patients diagnosed between 2005 and 2009 show a median overall survival of 8 months for all patients, increasing to 14 months for those who receive both surgery and chemotherapy (6). Despite the dismal prognosis of patients with mesothelioma irrespective of the type of therapy, studies have reported that long-term survival in pleural mesothelioma is possible in a subgroup of surgically treated patients (63, 64).

On imaging, pleural mesothelioma often manifests as diffuse, circumferential pleural thickening, frequently with invasion into the diaphragm, pericardium, chest wall, and mediastinum. Initial presentation is pleural effusion with or without pleural thickening in majority of cases, rarely presents as focal pleural mass with chest wall invasion. Epithelioid mesothelioma typically presents with more homogeneous, sheet-like growth, while biphasic and sarcomatoid subtypes display more asymmetric, focal nodular, and infiltrative patterns. On computed tomography (CT), classic findings include irregular pleural thickening, nodularity, volume loss, and associated pleural effusions. However, CT is often limited in accurately characterizing invasion and tumor margins, particularly in the setting of chest wall or diaphragmatic invasion (65). Consequently, magnetic resonance imaging (MRI), especially with diffusion-weighted imaging (DWI) and perfusion sequences, plays an increasingly valuable role in local staging and preoperative evaluation.

DWI MRI provides quantitative information about tumor cellularity via computation of apparent diffusion coefficient (ADC) values. Gill et al. have shown that ADC values correlate with histologic subtype and disease aggressiveness. We derived using two or more b-values, typically including b = 0–50 s/mm² and b = 450 and 1,000 s/mm². ADC maps are generally generated using a mono-exponential model on the scanner console. Regions of interest (ROIs) were manually drawn on the solid components of pleural tumor on co-registered post contrast gradient echo images, with explicit exclusion of necrotic, cystic, or hemorrhagic areas. Epithelioid tumors typically demonstrate higher ADC values (mean ~1.31 × 10^−3 mm²/s), while biphasic and sarcomatoid mesotheliomas show significantly lower values (~1.01 and 0.99 × 10^−3 mm²/s, respectively), reflecting increased cellularity and decreased extracellular matrix (66, 67). In biphasic tumors, DWI may help estimate the proportion of sarcomatoid components, with ADC values <0.94 × 10^−3 mm²/s suggesting a more aggressive behavior and poorer outcomes (68). As such, DWI is increasingly recognized as a noninvasive imaging biomarker for subtype differentiation, prognostication, and guiding biopsy.

For diffusion-weighted MRI, multiple studies have demonstrated significantly lower ADC values in malignant pleural mesothelioma compared with benign pleural disease. Reported mean ADC values for malignant pleural mesothelioma typically range from approximately 0.9–1.3 × 10^−3^ mm²/s, whereas benign pleural thickening and inflammatory pleural disease generally show higher values, often >1.4–1.6 × 10^−3^ mm²/s. Several single-center studies have proposed ADC thresholds in the range of 1.1–1.2 × 10^−3^ mm²/s for differentiating malignant from benign pleural processes, with reported sensitivities and specificities commonly exceeding 80%. However, these thresholds have primarily been derived from retrospective cohorts and remain not yet externally validated, limiting their current clinical generalizability. However, ADC values are used to assess response to targeted therapies in several tumors including Pleural Mesothelioma.

Perfusion MRI and FDG PET/CT further expand the functional imaging toolkit for mesothelioma. Perfusion MRI provides metrics of tumor vascularity (e.g., K^trans, Vp) which are linked to angiogenesis and may inform treatment planning, particularly in antiangiogenic strategies (69). FDG PET/CT offers critical insights into metabolic activity and is more accurate than CT alone for detecting nodal and distant metastatic disease. High standardized uptake values (SUVmax), commonly seen in biphasic and sarcomatoid tumors, correlate with worse survival. PET/CT also improves detection of mediastinal, supraclavicular, and abdominal nodal metastases, as well as extrathoracic spread to organs such as the liver, adrenal glands (69). Combined with MRI, PET/CT contributes to a more complete assessment of disease extent and guides surgical eligibility and multimodality treatment planning. For FDG PET/CT, quantitative metabolic parameters including SUVmax, metabolic tumor volume (MTV), and total lesion glycolysis (TLG) have been associated with tumor aggressiveness and clinical outcomes. SUVmax thresholds in the range of 3.5–5.0 have been reported for distinguishing malignant pleural mesothelioma from benign pleural disease, while higher baseline SUVmax (>10), MTV, and TLG values have been associated with worse overall survival and progression-free survival. Among these, volumetric PET metrics (MTV and TLG) appear to demonstrate stronger prognostic performance than SUVmax alone in several studies. Nonetheless, these PET biomarkers have largely undergone single-institution or limited multicenter validation, and standardized cutoff values have not been universally adopted.

## Microscopic heterogeneity of mesothelioma

Mesothelioma can have different histologic types which are identifiable when tumor tissue is visualized under the optic microscope. The primary histologies are epithelioid, sarcomatoid, and biphasic (4) which form the basis of mesothelioma classification and have important prognostic significance (27, 70, 71).

Regardless of the histologic type, pleural mesothelioma can display several morphologic variants and patterns based on its cytologic, architectural, and stromal features (72). The tumor cells of epithelioid mesothelioma are usually ovoid-round, or polygonal with eosinophilic cytoplasm and cohesive; other patterns include clear-cell, signet ring-cell, rhabdoid, deciduoid, and small-cell (73–75). Tumor cells are arranged in diverse architectural patterns that include tubulopapillary, trabecular, solid, acinar, micropapillary, or adenomatoid. The morphologic patterns of sarcomatoid mesothelioma include elongate tumor cells with conventional/spindle cell, desmoplastic (76, 77), and lymphohistiocytoid (78–80). A subset of sarcomatoid mesothelioma exists which exhibits heterologous differentiation with osteosarcomatous, chondrosarcomatous, or rhabdomyosarcomatous elements (77). The recognition of these diverse histotypes has largely focused on diagnostic considerations i.e. avoiding a tumor mimic and optimizing the application of immunohistochemistry to refine an accurate diagnosis.

Due to the inter-tumoral and intra-tumoral morphologic heterogeneity, classification based on histologic type may be difficult. In some cases, it may be problematic to distinguish between neoplastic epithelioid tumor and reactive mesothelial hyperplasia, or alternatively neoplastic spindled tumor cells (sarcomatoid mesothelioma) and reactive stromal fibroblasts. Similarly, the distinction between biphasic mesothelioma and epithelioid mesothelioma with prominent cellular spindled stroma can be challenging (81). Furthermore, there is considerable interobserver variability in the recognition of histologic types, even among mesothelioma expert pathologists, with the lowest interobserver agreements in biphasic mesotheliomas (81–83). Accurate classification of mesothelioma is nevertheless important, given the prognostic significance and therapeutic alternatives for different histologic types.

Due to intratumoral heterogeneity, the accuracy of histologic classification varies with the extent of tissue sampling (71, 84, 85). In a study comparing the concordance between histologic types in initial biopsies with subsequent resections, the accuracy of typing increases with a higher number of biopsies (71). While sarcomatoid histology in biopsies is highly predictive of sarcomatoid histology in resections, epithelioid histology in biopsies is not entirely specific and is changed to biphasic or sarcomatoid types in resections in up to 20% of patients (71). The initial thoracoscopic biopsies that had concordant diagnoses with surgical resections were sampled with a median number of 3 tissue blocks (71).

It is now recognized that the accurate pathologic diagnosis of mesothelioma reporting requires extensive work-up, including adequate sampling, marker studies, molecular analysis and external expert review to define primary tumor subtyping, architectural variant, nuclear grade (for epithelioid histotype only), extent of patterns, stage of disease, and characterization of other significant pathology characteristics with clinical and therapeutic significance.

## Genetic characteristics of mesothelioma

Most diffuse mesotheliomas lack oncogenic kinase mutations and in contrast have genetic alterations including single nucleotide, copy number or structural alterations that perturb the functions of tumor suppressor genes and chromatin regulating genes. Furthermore, the mutation burden of mesotheliomas is extremely low (typically fewer than two mutations per megabase) and there are relatively few recurrent mutations (86).

Diffuse mesothelioma is typically characterized by multiple copy number gains and losses throughout the genome (87), including recurrent loss of *BAP1*, *CDKN2A*, *MTAP*, and/or *NF2* in a subset of tumors (88, 89). Fluorescence *in situ* hybridization assays could investigate minute interstitial gene deletions in *BAP1*, *CDKN2A*, and *NF2*, which can distinguish mesothelioma from benign reactive mesothelial proliferations (90). In addition, high-density array comparative genomic hybridization can be used to detect non-contiguous minute deletions of relevant genes such as *BAP1* in diffuse mesotheliomas (91).

In addition to copy number alterations, recurrent somatic mutations present in diffuse mesotheliomas have been identified by whole-genome, whole-exome, and/or transcriptome sequencing (92–95). These somatic alterations affect multiple cellular pathways, including cell cycle/DNA repair (*BAP1*, *TP53*, *CDKN2A*, *CDKN2B*), Hippo signaling (*NF2*, *LATS1*), RNA machinery (*DDX3X*, *SF3B1*), and histone regulation (*SETD2*, *SETDB1*). Complex chromosomal alterations including chromothripsis and chromoplexy have also been detected in diffuse mesotheliomas (96).

## Genomic heterogeneity of mesothelioma histotypes

In contrast to epithelioid mesothelioma, sarcomatoid mesothelioma has *BAP1* alterations less frequently (~50% *vs* ~20%), while *CDKN2A/MTAP* alterations are more common (~40% *vs* ~80%). The prevalence of distinctive genetic alterations including *BAP1*, *CDKN2A*, *MTAP*, and *NF2* in diffuse mesotheliomas of epithelioid, biphasic, and sarcomatoid histotypes is variable (**Table 1**) and different between pleural, peritoneal, tunica vaginalis testis and pericardium (**Table 2**) (2, 97–100). These differences have been described in detail in a recent review (2) published by one of the authors and presented in **Tables 1** and **2**. While epithelioid and non-epithelioid mesotheliomas have a similar tumor mutational burden (97), *TERT* alterations (including promoter mutations) are more frequent in non-epithelioid histotypes (97, 110).

**Table 1:** Prevalence of select genetic alterations in mesothelioma of different histotype (97–100).

Histotype	*BAP1*	*CDKN2A*	*MTAP*	*NF2*
Overall (97, 98, 100)	44-53%	25-49%	27-34%	21-33%
Epithelioid (97, 99, 100)	51-59%	22-55%	28-45%	20-63%
Biphasic (97, 99, 100)	35-59%	39-89%	46-74%	22-74%
Sarcomatoid (99, 100)	17-25%	~80%	47-83%	50-83%

Adapted from reference^2^.

**Table 2:** Prevalence of select genetic alterations in mesothelioma of different primary site (88, 93, 94, 97–99, 101–109).

Primary site	*BAP1*	*CDKN2A*	*MTAP*	*NF2*
Pleura (88, 93, 94, 97–99, 108)	43-68%	48-69%	32-57%	33-74%
Peritoneum (97, 98, 101–103)	48-69%	8-26%	14-15%	19-27%
Paratesticular (104, 105)	0-29%	0-43%	unknown	66-71%
Pericardium (106, 107, 109)	~70%	~50%	~50%	~70%

Adapted from reference^2^.

Mesotheliomas with transitional pattern are characterized by sheet-like growth of cohesive plump elongated cells, which appear morphologically intermediate between epithelioid and sarcomatoid histotype; this has historically led to difficulty in histotype classification (81, 83). In transcriptomic profiling with unsupervised clustering analysis, transitional mesotheliomas cluster closely with sarcomatoid rather than epithelioid mesothelioma (111). Given its transcriptomic similarities to sarcomatoid mesothelioma and the poor prognosis, transitional pattern is currently classified as sarcomatoid. Furthermore, reticulin stain delicately outlines individual tumor cells in transitional mesothelioma, in contrast to the intense banding of individual cells in sarcomatoid mesothelioma and strapping of large cell clusters in epithelioid mesothelioma. Homozygous deletion of *CDKN2A* is present in ~70% of in transitional mesothelioma (111).

Mesotheliomas with pleomorphic pattern display prominent anaplasia, bizarre nuclei and have a poor prognosis (112, 113). By whole-exome sequencing analysis, pleomorphic mesotheliomas harbor recurrent alterations involving *CDKN2A*, *BAP1* and *NF2*, rarely *TP53* and *LATS1*/*LATS2*, similar to mesotheliomas of other histotypes and no distinctive, novel mutations unique to pleomorphic subtype are identified (114). Unlike transitional mesothelioma that has been reclassified as sarcomatoid based on transcriptomic data, pleomorphic mesothelioma appear genetically heterogeneous, and classification into single subtype remains problematic (114).

Other unusual histologies like pleomorphic rhabdoid cells or myxoid stroma, with high-grade cytology have been associated with mesotheliomas secondary to therapeutic radiation exposure for prior malignancy (26, 27),

## Immunohistochemistry for BAP1, MTAP, and merlin

As a consequence of *BAP1* mutations or copy-number loss, BAP1 expression shows aberrant pattern with complete loss of nuclear staining and is present in approximately 40-60% of mesotheliomas (115–119). The presence of cytoplasmic staining alone without nuclear staining present in a subset of tumors also indicates the presence of *BAP1* alterations (118, 119). BAP1 immunohistochemistry has become a helpful diagnostic adjunct in the evaluation of mesothelial lesions. In addition to the evaluation of BAP1, immunohistochemistry for MTAP and merlin (encoded by *NF2*) has been used in the diagnostic evaluation, with loss of cytoplasmic MTAP staining and loss of merlin staining present in a subset of mesotheliomas (99, 115–117, 120–125). The prevalence of aberrant BAP1, MTAP, and merlin expression among mesotheliomas of different histotypes was recently summarized in a comprehensive review (2) and listed in **Table 3** (99, 116, 118, 119, 121, 122, 125, 126). Similarly, the prevalence of aberrant BAP1, MTAP, and merlin expression among mesotheliomas of different primary sites is summarized in **Table 4** (88, 90, 99, 101–106, 118, 121, 127).

**Table 3:** Prevalence of aberrant immunohistochemical expression of BAP1, MTAP, and merlin in mesothelioma of different histotype (99, 116, 118, 119, 121, 122, 125, 126).

Histotype	Nuclear loss of BAP1	Cytoplasmic loss of MTAP	Complete loss of merlin
Overall (99, 116, 118)	54-66%	~50%	~50%
Epithelioid (99, 116, 118, 119, 121, 125)	55-70%	35-65%	35-41%
Biphasic (99, 116, 118, 119)	47-77%	~60%	~70%
Sarcomatoid (99, 116, 118, 119, 122, 125, 126)	10-36%	61-83%	39-67%

Adapted from reference^2^.

**Table 4:** Prevalence of aberrant immunohistochemical expression of BAP1, MTAP, and merlin in mesothelioma of different primary site (88, 90, 99, 101–106, 118, 121, 127).

Primary site	Nuclear loss of BAP1	Cytoplasmic loss of MTAP	Complete loss of merlin
Pleura (88, 99, 118, 121)	54-67%	46-67%	~50%
Peritoneum (90, 101–103, 127)	50-85%	17-19%	unknown
Paratesticular (104, 105)	20-22%	0-17%	unknown
Pericardium (106)	~70%	~70%	~70%

Adapted from reference^2^.

Comprehensive correlative studies between molecular findings by hybrid-capture next-generation sequencing (NGS) panel including regions for *BAP1, CDKN2A/MTAP, NF2*, and *TP53*; and diagnostic immunostains for BAP1, MTAP, merlin, and p53, respectively have recently been published, also exploring a re-evaluation of current ancillary testing algorithms (99).

## Other mesothelial lesions and benign mesothelial proliferations

In addition to diffuse mesothelioma, the World Health Organization (WHO) recognizes additional types of mesothelial lesions: (1) localized mesothelioma, (2) well-differentiated papillary mesothelial tumor, and (3) adenomatoid tumor (3, 13).

*Localized pleural mesothelioma* is microscopically identical to diffuse mesothelioma, although it is radiographically and grossly solitary and circumscribed (128–130). This unusual disease distribution may result in alternate clinical disease considerations such as metastases, lung carcinoma, or chest wall sarcoma. Genetically, localized pleural mesothelioma includes three groups (*BAP1*-mutant, *TRAF7*-mutant, and near-haploid), with similarities but also differences from diffuse malignant pleural mesothelioma (131).

*Well-differentiated papillary mesothelial tumor (WDPMT)*, often an incidental finding in the peritoneum of women, can occur in the pleura (132) and is genetically characterized by recurrent mutations in *TRAF7* or *CDC42* (133). Infrequently, well-differentiated papillary mesothelioma shows back-to-back papillae with foci of invasion (invasive foci) (134), morphologically mimicking diffuse malignant mesothelioma. Furthermore, distinction between a malignant mesothelioma with prominent papillary surface projections and well-differentiated papillary mesothelioma can be challenging, particularly in small superficial biopsies. WDPMT shows retained nuclear expression for BAP-1 and this allows its distinction from papillary variant mesothelioma-*in situ* (133, 135).

*Adenomatoid tumor* primarily affects the genital tract but rarely can involve the pleura; recurrent mutations in *TRAF7* have been described in adenomatoid tumors of genital-type (136).

## Distinctive mesotheliomas with unique genetic features

Random stochastic genetic mutations also cause mesothelioma - including structural gene rearrangements involving oncogenes such as Ewing sarcoma breakpoint region 1 (*EWSR1*) gene or fused in sarcoma (*FUS*) gene (35), and anaplastic lymphoma kinase (*ALK*) gene rearrangements, recently described in a study regarding a cohort of young women with peritoneal mesothelioma (36).

Furthermore, inherited mutations such as germline alterations in *BAP1* and in other tumor suppressor genes have been implicated in the development of mesothelioma in a subset of approximately 12%-16% of patients (37–42).

## Germline mutations in diffuse mesotheliomas

In a proportion of subjects with mesothelioma, approximately 12-16% have specific pathogenic variant germline mutations which are implicated in their development of cancer (37–42). It is clinically important to identify such cases for both the individual patients and their families since it has critical implications for therapeutic strategies and genetic counseling (37–40, 137–143). Mesotheliomas arising in the setting of inherited genetic syndromes and/or pathogenic variant germline mutations are more common in patients of young age (<50years), in mesothelioma of the peritoneum (25%) versus pleura (7%), in patients with no identifiable asbestos exposure, low-grade epithelioid histotype with high inflammatory tumor microenvironment and either a setting of concomitant personal or family cancers (38).

*BAP1* has emerged as one of the genes frequently mutated in the germline setting. In germline *BAP1* inactivation syndrome, patients and their family members can present with a constellation of mesothelioma, uveal melanoma, renal cell carcinoma, and/or other tumors (41). To date, more than 200 families with germline *BAP1* mutations have been described (144, 145). In addition to the development of invasive mesothelioma, the presence of pathogenic variant germline *BAP1* mutation may confer increased risk to developing mesothelioma *in situ*; for instance, mesotheliomas *in situ* involving bilateral pleural and peritoneal cavities has been reported in a patient harboring pathogenic variant germline *BAP1* mutation.

*BRCA2 germline mutations and mesothelioma.* Recent studies employ whole exome sequencing (WES) and whole genome sequencing (WGS) of mesothelioma tumor samples to identify germline pathogenic variants. A recent study (137) evaluated the incidence of germline pathogenic variants in patients with mesothelioma, who were not selected for their family history of mesothelioma or cancer, using WES or WGS. In this retrospective cohort of 44 consecutive patients with mesothelioma, 36% of the patients had a germline pathogenic variant in various genes, including *BRCA2*. Associations between *BRCA2* germline mutations and mesothelioma have been also previously reported by Betti 2017 (141), Panou 2018 (38), Hassan 2019 (37), Guo 2020 (39), Srinivasan 2021 (146), Cheung 2021 (147). Furthermore, a recently-published clinical trial (148) demonstrated promising results in patients with solid tumors that had *BRCA1/2* mutations. In this trial, one patient with peritoneal mesothelioma induced by *BRCA2* germline mutations achieved partial tumor response (148).

Germline mutations in other tumor suppressor genes have been identified in patients with pleural and/or peritoneal mesotheliomas. In addition to *BAP1*, this list of genes includes *CHEK2*, *CDKN2A*, *ATM*, *ATR*, *RECQL4*, *BRCA1*, *TP53*, *PTEN*, *NF2*, *MLH1*, and *MSH6*, among others (38, 39, 147, 149–152). These genes primarily pertain to the cell cycle regulation and DNA repair pathways.

There is an evolving debate in relation to the role of asbestos in subjects with inherited genetic syndromes and mesothelioma i.e. are the subjects susceptible to developing cancers or are they susceptible to the effects of asbestos exposure? The fact that there is an observed female preponderance in these inherited genetic cases *and* the fact that half of the tumors were peritoneal in origin argue against the position that such subjects are sensitized to low-level asbestos exposures. It is known that most asbestos-induced mesotheliomas are pleural, arising in elderly men following workplace exposures to commercial amphibole asbestos. It would be expected that asbestos carcinogenesis augmented by a genetic predisposition (so-called gene-environment interaction) should maintain this ratio (pleural:peritoneal; 9:1) but this is not seen, neither is the male:female preponderance; indeed, if the presumption is that these patients had low-level inhalational asbestos exposures, it is hard to explain how they could develop more peritoneal than pleural mesotheliomas – since inhaled biopersistent asbestos fibers would still interact with pleural cells before translocating to the peritoneum. Moreover, gene-environment interaction does not explain the observation that women with inherited genetic syndromes are equally affected (not less) since they typically do not experience such workplace exposure to commercial amphibole asbestos necessary to induce peritoneal mesothelioma. It is clear that mesotheliomas arising in the setting of inherited genetic syndrome can and do arise via mechanisms unrelated and independent of asbestos in most settings (153). Furthermore, it has been shown that asbestos does not directly interact with *BAP-1* mediated cellular mechanisms (153).

Some heterozygous germline mutations in genes like *BLM* have been suggested to play a role in altering mesothelioma development in patients with prior asbestos exposure, but hypotheses of how these particular germline variants interact with environmental exposure such as asbestos in the pathogenesis of mesothelioma in a given patient remain complex, unclear and speculative (154).

While somatic mutations in *NF2* are present in a subset of mesotheliomas, it remains unclear whether the presence of germline *NF2* mutation alone are associated with a significant risk to developing mesothelioma (152), since a second-hit event is required for biallelic inactivation (155).

## Uncommon alterations in diffuse mesotheliomas

A small subset of mesotheliomas harbor unusual somatic alterations such as genomic near-haploidization, *ALK* rearrangement, *EWSR1*::*ATF1* or *FUS*::*ATF1* fusion, *EWSR1*::*YY1* fusion which are discussed below.

### Mesothelioma with genomic near-haploidization

Genomic near-haploidization is present in approximately 3% of diffuse mesotheliomas in both the TCGA-ICGC cohort and a comprehensive genomic profiling database (93, 97, 156). Mesotheliomas with genomic near-haploidization have been reported involving the pleura or the peritoneum in patients with a wide age range. Genomic near-haploidization can be detected by either karyotypic analysis using fresh samples or next-generation or whole-genome sequencing on formalin-fixed or frozen samples (93, 97, 102, 103). Genomic near-haploidization in mesotheliomas is characterized by extensive loss of heterozygosity throughout the genome except the constant retention of chromosomes 5 and 7 (93, 97, 102, 103). In mesotheliomas with near-haploidization *BAP1* alterations are uncommon and instead mutations in *TP53*, *SETDB1*, and *NF2* have been commonly reported (93, 97). Microscopically, near-haploid mesotheliomas have no distinctive morphology and can be epithelioid, biphasic, or sarcomatoid, mesotheliomas with *VHL* inactivating mutations and genomic near-haploidization are epithelioid with prominent clear-cell histology (157).

### Mesothelioma with *ALK* rearrangement

*ALK*-rearranged mesotheliomas are rare, with series (36, 158–160) and case reports described recently in the medical and scientific literature (161–164). Particularly, *ALK*-rearranged mesotheliomas have a wide age distribution ranging from children (159–161) to adults (36), involve usually the peritoneum, and demonstrate epithelioid or biphasic histology with ALK protein over-expression. Since *ALK* rearrangement is present in diverse tumor types with epithelioid or spindled morphology that may overlap with mesothelioma, in order to diagnose *ALK*-rearranged mesothelioma, it is critical to confirm that the tumor cells have mesothelial differentiation. *ALK*-rearranged mesotheliomas can express PAX8 (159), which could diagnostically mimic Mullerian or peritoneal carcinomas. ALK testing by immunohistochemistry using sensitive antibody clones (such as D5F3 and 5A4, but not ALK1 antibodies) (165) is considered a reliable surrogate of *ALK* rearrangement in non-small cell lung carcinomas (166), but ALK over-expression can be present in some mesotheliomas (167) [and various other tumors (168– 170)] that do not harbor *bona fide ALK* rearrangement. To confirm *ALK* rearrangement in mesotheliomas, additional testing using next-generation sequencing (NGS) and/or fluorescence *in situ* hybridization assays is advocated. In contrast to non-small cell lung carcinomas where *EML4* is the most common fusion partner to *ALK*, *STRN* is the most frequent fusion partner to *ALK* in *ALK*-rearranged mesotheliomas (36, 158, 159, 163, 164, 171, 172). Treatment of patients with *ALK*-rearranged peritoneal mesothelioma using first or second generation ALK-targeted inhibitors (171, 172) has been successfully used in the clinical setting, and in a rare case, emergence of resistance to ALK-targeted therapy (172), has also been reported. Among patients with peritoneal mesothelioma and *ALK* rearrangement, three patients with an exon 3 *STRN*-exon 20 *ALK* fusion were reportedly treated with ALK inhibitors (173). One was a 9-year-old female treated with crizotinib whose tumor did not respond (159), a second patient was a 24-year-old female treated with crizotinib with a partial response lasting for only 4 months (172), and the other was a 13-year-old female treated with ceritinib with a partial response ongoing over 3 months (171). Given the potential therapeutic value of ALK inhibitors, the NCCN Guidelines recommend broad molecular tumor profiling for peritoneal mesothelioma (60). Another comprehensive study describing mesothelial tumors associated with fusions identified seven patients with *ALK* rearrangements, and only one treated with the anaplastic lymphoma kinase inhibitor alectinib, but offered no information regarding the tumor response (174).

Aside from *ALK* rearrangement, *TPM3*::*NTRK1* fusion has been reported in one pleural mesothelioma, however the clinicopathologic details were missing (175). Based on fluorescence *in situ* hybridization studies, no mesotheliomas with *ROS1*, *RET*, or *MET* rearrangement have been identified to date (176, 177).

### Mesothelioma with *ATF1* rearrangement

*ATF1* rearrangement, characterized by *EWSR1*::*ATF1* or *FUS*::*ATF1* fusion, has been reported in some mesotheliomas in limited case series or reports (35, 178, 179). *ATF1*-rearranged mesotheliomas present typically in the peritoneum of adolescents or young adults, display epithelioid histology, and have intact BAP1 expression (35, 178, 179). Since *ATF1* rearrangement is present in diverse other tumor types (clear cell sarcoma, angiomatoid fibrous histiocytoma, and hyalinizing clear cell carcinomas), it is important to confirm that the tumor cells are of mesothelial differentiation. In addition to immunohistochemical evaluation to establish mesothelial differentiation, methylation profiling can be employed, which shows the apparent clustering of *ATF1*-rearranged mesotheliomas with conventional mesothelioma (180). Nonetheless, the relationship between *ATF1*-rearranged mesotheliomas and other cytokeratin-positive peritoneal neoplasms characterized by *EWSR1* or *FUS* fused to other members of CREB-transcription factors, such as *CREM* and *CREB1* (181, 182) remains unclear.

### Mesothelial neoplasms with *NR4A3* fusions

These fusions recognized in the scientific literature characterize a distinctive peritoneal mesothelial neoplasm of uncertain biological potential with extensive adenomatoid/microcystic morphology (183). This morphology is rare amongst conventional forms of diffuse mesothelioma. Such specific genetic fusions should be considered likely if this morphology is seen in a peritoneal mesothelial neoplasm with retained BAP-1, retained CDKN2A, low proliferation fraction (Ki-67 1-2%) and PAX-8 expression. It is important to identify and test for such fusions as these tumors have a reported indolent clinical behavior.

### Mesothelioma with *EWSR1::YY1* fusion

*EWSR1*::*YY1* fusion is uncommon in mesothelioma, with 5 cases described to date (180, 184). *EWSR1*::*YY1* fusion positive mesotheliomas can be localized or diffuse, involve the peritoneum of both sexes in middle-aged adults, and often present incidentally. Histologically, *EWSR1*::*YY1* fusion positive mesotheliomas demonstrate epithelioid histology, have trabecular or solid architecture and have intact BAP1 expression. While the two *EWSR1*::*YY1* fusion positive mesotheliomas analyzed by methylation profiling cluster together with mesotheliomas with conventional genetics and *ATF1*-rearranged mesotheliomas, evaluation of methylation status and how *EWSR1*::*YY1* fusion mesotheliomas differ from other mesotheliomas remains unclear due to limited number of cases (180).

In summary, amongst gene fusion associated mesothelioma/mesothelial neoplasms, almost all arose in the peritoneal setting, had epithelioid morphology and arose in young subjects with no history of asbestos exposure (35, 36, 158, 159, 178–180, 183, 184). The development of mesothelioma in conjunction with specific gene fusions in subjects of an age below the shortest latent period associated for asbestos-induced mesothelioma provides strong evidence that the tumor has arisen independent of asbestos. Furthermore, specific cytogenetic alterations are identified in a variety of neoplasms including a variety of different soft tissue tumors, melanomas, salivary gland carcinomas. All these tumors with EWSR1 gene fusions arise unrelated to asbestos. The specific *EWSR1-NR4A3* fusion identified in a subset of peritoneal epithelioid mesothelial neoplasms was also identified in extraskeletal myxoid chondosarcoma and acinic salivary carcinoma of salivary gland (185–188), tumors with no evidence of asbestos exposure.

## Diagnostic and clinical implications of molecular testing in mesothelioma

Molecular methods that are currently used in diagnostic mesothelial proliferations include: fluorescence *in situ* hybridization (FISH) assays (testing for characteristic loss of relevant genes such as *BAP1*, *NF2*, *CDKN2A*, and *MTAP*), single-nucleotide polymorphism (SNP) microarrays (testing for genome-wide copy number variations), next-generation DNA- or RNA-based sequencing assays (testing for single-nucleotide, structural, or other variants in the targeted genome), and DNA methylation profiling (testing for gene expression patterns) (189, 190).

The utility of molecular testing in the workup of patients with mesothelioma has three major applications: diagnostic, prognostic, and predictive (2).

1. First, the diagnosis of mesothelioma can be challenging, and molecular testing can assist in challenging circumstances where the histologic and immunophenotypic features are equivocal. Detection of relevant mutations by next-generation sequencing or gene loss by FISH would support neoplasia when differentiating between mesothelioma/mesothelioma *in situ* and reactive mesothelial proliferation - or between sarcomatoid mesothelioma and chronic fibrosing pleuritis. In the distinction between mesothelioma and other malignancies, next-generation sequencing can reveal mutations that may suggest tumor origin (191).
2. Second, molecular testing can provide prognostic information by incorporating mutation status and correlation with well-annotated clinical and genomic databases. For instance, the presence of *TP53* mutation has been associated with worse prognosis in pleural mesotheliomas (92). In addition to single gene-based prediction, more elaborate systems may utilize next-generation sequencing data with machine learning for prognostication (192). Transcriptomic analysis has also been used to generate prognostic gene signatures in pleural and peritoneal mesotheliomas (193).
3. Third, molecular testing can provide predictive information on how mutation status may affect outcome and clinical decision-making. For instance, patients with pleural or peritoneal mesothelioma harboring germline *BAP1* mutations are known to have a longer overall survival (151). Patients with pleural mesothelioma harboring germline *BAP1* mutations have reportedly improved response to platinum chemotherapy (37). In addition to germline *BAP1* status, patients harboring germline mutations in cancer-predisposing genes in general are more likely to survive longer after treatment with durvalumab and platinum-pemetrexed (194). Additionally, candidate factors to predict immunotherapy response in mesothelioma include *BAP1* haploinsufficiency status (195), persistent tumor mutational burden (196), and tumor junction burden with analysis of antigen presentation (197), among others. Of note, inactivated *BAP1* status is considered as one of the inclusion criteria in the clinical trials on mesothelioma using EZH2-targeted inhibitor tazemetostat (198). Also, as discussed earlier, the presence of oncogenic rearrangements such as *ALK* fusion in rare mesotheliomas presents a therapeutic target. Finally, in a recent analysis, molecular factors including tumor ploidy, adaptive immune response, and CpG island methylation profile (CIMP) can be incorporated in conjunction with tumor cell morphology to generate a morpho-molecular classification of diffuse mesotheliomas to improve prognostication and potentially therapy response prediction (95, 199).

## Conclusions

Here we provide an overview of the recent discoveries on the genetics and pathogenesis of mesothelioma, as well as the implications of these findings on the diagnostic workup. We attempted to distinguish between strong correlative analyses with implications for clinical decision-making and associations that remain insufficiently described or poorly understood. Limitations are also induced by the low incidence of mesothelioma globally, that would make statistical analyses have a low degree of certainty (weak associations). We stated that certain genotype-phenotype associations remain unclear or rather speculative. In other instances, as in the setting of *ALK*-rearranged mesothelioma we present proof of response (partial or complete), even if data is anecdotal, but we make the point that given the catastrophic outcome of these patients with such an aggressive disease, recommendations were incorporated in clinical national guidelines (200) in order to attempt treatment with targeted therapies.

The emergence of modern laboratory technologies such as advancing next generation sequencing and evolving novel immunohistochemistry markers advanced our understanding on the molecular diversity of diffuse mesothelioma and identification of distinct mesothelial neoplasms and led to considerable changes in our diagnostic practice, including the application of immunohistochemical markers such as BAP1, MTAP, and merlin (NF2) that are surrogates for their mutation status (99, 115–117, 120–125). In patients with unusual family cancer histories, or young patients and/or those without significant asbestos exposure, unusual mesothelioma genetics such as germline mutations, *ALK* rearrangement, near-haploidization and *ATF1* rearrangements should be considered. Some of the emerging categories of mesothelioma can be difficult to confirm with conventional immunohistochemistry alone and require molecular testing. There is no doubt that further advancements will uncover novel mechanistic insights into molecular pathogenesis, improve prognostication and introduce new hopeful treatment options for patients with such a devastating disease.
